# Supplementary material for: A spatio-temporal model to reveal oscillator phenotypes in molecular clocks: Parameter estimation elucidates circadian gene transcription dynamics in single-cells
Source: PLoS Comput Biol. 2021 Dec 17;17(12):e1009698. doi: 10.1371/journal.pcbi.1009698 (PMC8719734; doi:10.1371/journal.pcbi.1009698)
Supplement: S1 Text — Stability criteria of macroscopic rate equation. Derivation of stability criteria used to calculate robustness of oscillations. Likelihood approximation of TTFL model. Equations and description of implementation for filtering procedure used to approximate the likelihood of the TTFL model. Remarks on studying explant SCN. Description of tissue preparation. Fig A. Spatial distribution of residual periodicity. Model fit evaluated using spectral bootstrap of residuals. Fig B. Average signal in subset of pixels by V^θ cut-off. Visualization of observed oscillations (individual pixels and aggregate) around the edge of the SCN as given by V^θ. Fig C. Period, phase and amplitude for three replicates of Cry1:luc. Results of descriptive spectral analysis of Cry1-luc replicates 1–3. Fig D. Inferred Per2::luc oscillator robustness in VIP knock-out tissue. Spatial distribution of robustness measure V^θ from imaging data of Per2 expression in VIP-null SCN. Fig E. Differential response to large negative perturbation. Resulting oscillations and phase shift distributions under variation of simulation study. Fig F. Spatial distribution of posterior means. Spatial distribution of posterior means for degradation rate (μ), light scaling constant (κ) and measurement error SD (ση). (PDF) [file pcbi.1009698.s001.pdf]

S1 Supporting Information
A spatio-temporal model to reveal oscillator phenotypes in molecular clocks

Måns Unosson[1], Marco Brancaccio[2], Michael Hastings[3], Adam M. Johansen[1], and Bärbel Finkenstädt[1,4,*]

[1] *Department of Statistics, University of Warwick, Coventry UK.*
[2] *UK Dementia Research Institute at Imperial College London, Department of Brain Sciences, Faculty of Medicine, London, UK*
[3] *MRC Laboratory of Molecular Biology, Division of Neurobiology, Cambridge, UK.*
[4] *The Zeeman Institute for Systems Biology & Infectious Disease Epidemiology Research, University of Warwick, Coventry, UK.*
[*] *b.f.finkenstadt@warwick.ac.uk*

December 12, 2021

1 Stability criteria of macroscopic rate equation.

To obtain stability criteria for the macroscopic rate equation, given by

$$\frac{dX(t)}{dt} = \frac{R}{1 + \left(\frac{\int_{-\infty}^{t} X(s) g_{p,\alpha}(t-s) ds}{K}\right)^n} - \mu X(t) \tag{1}$$

we make use of the linear chain trick [1] to obtain the following system of ODEs for $X_0(t) := X(t)$ and intermediate states X_i,

$$\begin{aligned} \frac{dX_0(t)}{dt} &= \frac{R}{1 + \left(\frac{X_p(t)}{K}\right)^n} - \mu X_0(t) \\ \frac{dX_i(t)}{dt} &= a(X_{i-1}(t) - X_i(t)), \;\; i = 1, ..., p. \end{aligned} \tag{2}$$

which is a monotone cyclic feedback system (MCFS) [2], i.e. flows through the system are unidirectional as dX_i/dt is monotonically forced by X_{i-1}. By Theorem 4.1 of ref. [2] the omega limit sets of solutions to a MCFS with negative feedback, and $\det(-J(x^*)) > 0$, where J is the Jacobian of the system and x^* is the unique fixed point $X_i = x^*$ s.t. $dX_i/dt = 0$ for $i = 0, ..., p$, are either non-constant periodic orbits or the unique fixed point. To show that $\det(-J(x^*)) > 0$, we make

use of the sparse structure of the (negative) Jacobian,

$$-J(x^*) = \begin{pmatrix} \mu & 0 & \dots & 0 & -f_0'(x^*) \\ -a & a & 0 & \dots & 0 \\ 0 & -a & a & \ddots & \vdots \\ \vdots & \ddots & \ddots & \ddots & 0 \\ 0 & 0 & 0 & -a & a \end{pmatrix}, \tag{3}$$

and note that $\det(-J(x^*))$ can be decomposed as

$$\det(-J(x^*)) = \mu \det(A) - (-1)^p f_0'(x^*) \det(-A^\intercal) \tag{4}$$

where A is the $p \times p$ bidiagonal sub-matrix obtained when deleting the first row and column of $-J(x^*)$. The determinant of A is a^p, and hence we obtain

$$\det(-J(x^*)) = (\mu - f_0'(x^*))a^p > 0 \tag{5}$$

for μ, $a > 0$ and $f_0'(x^*) < 0$. To determine whether a given set of parameters imply limit cycle or damped dynamics we examine the real part of the dominant eigenvalue of $J(x^*)$, where a positive real part implies a limit cycle and a negative real part (and non-zero imaginary part) imply damped oscillations. To calculate the posterior probability of a limit cycle the matrix J is constructed for the thinned MCMC output and the real part of the largest eigenvalue is evaluated using an indicator function that takes the value 1 if the eigenvalue has positive real part and 0 if negative. The mean of the resulting chain of indicators is reported as the posterior probability of a limit cycle. Note however that the stability result is derived under $p \in \mathbb{N}$, while the MCMC samples of p take values in $\mathbb{R}$. To approximate the Jacobian we device a rounding scheme for p and a that preserves the dispersion of the delay distribution, given by

$$\begin{aligned} p' &= \lfloor p \rceil \\ a' &= \sqrt{a^2 \frac{p'}{p}}, \end{aligned} \tag{6}$$

where $\lfloor \cdot \rceil$ denotes rounding to nearest integer. To investigate sensitivity to the rounding we repeated the analysis for $\lfloor p \rfloor$ and $\lceil p \rceil$ and conclude that the choice of rounding scheme did not impact the findings.

2 Likelihood approximation of TTFL model.

In this section we give a description of the implementation of the likelihood approximation using the extended Kalman-Bucy filter. Consider a time discretization of Eq (1-2) of the main text, given

by

$$\begin{aligned}
Y_t &= \kappa \frac{\Delta t}{\delta t} \sum_{s=t-\Delta t}^{t} X_s + \eta_t = F X_{t-\Delta t:t} + \eta_t \\
X_t &= X_{t-\delta t} + \delta t \left[\frac{R}{1+\left(\frac{d(X_t)}{K}\right)^n} - \mu X_{t-\delta t} + \left(\frac{R}{1+\left(\frac{d(X_t)}{K}\right)^n} + \mu X_{t-\delta t} \right)^{1/2} Z_t \right] \\
d(X_t) &= \sum_{s=t-\tau_{\max}}^{t-\delta t} X_s \tilde{g}_{p,a}(t-s+\delta t/2)\delta t \\
\eta_t &\sim \mathcal{N}(0, \sigma_\eta^2) \\
Z_t &\sim \mathcal{N}(0, 1),
\end{aligned} \tag{7}$$

where $\tilde{g}_{p,a}$ is the gamma delay density truncated at $\tau_{\max}$. Assume there exists an optimal estimate of the normal distribution of the initial state condition given observations $\pi(x_{0:\tau_{\max}}|y_{0:\tau_{\max}})$ with mean vector $\rho_{0:\tau_{\max}}$ and covariance matrix $P_{0:\tau_{\max}}$. In practice we obtain these by scaling initial data by the current κ. By Taylor expanding the discretized state transition equation about the mean vector $\rho_{t-\tau_{\max}:t}$ at each iteration and dropping terms of order $(\delta t)^2$, we obtain the following equations for the mean and covariance

$$E[X_{t+\delta t}|y_{0:t}] = \rho_{t+\delta t} \approx \rho_t + \delta t \left(\frac{R}{1+\left(\frac{d(\rho_t)}{K}\right)^n} - \mu \rho_t \right) \tag{8}$$

and

$$\begin{aligned}
\mathrm{Cov}[X_{t-\tau_{\max}+\delta t:t+\delta t}|y_{0:t}] = P_{t+\delta t} \approx & P_t + \delta t[\mu \rho_{t-\tau_{\max}+\delta t:t+\delta t} P_t + P_t^\intercal \mu \rho_{t-\tau_{\max}+\delta t:t+\delta t}^\intercal] \\
& + \delta t \left[- \frac{nR\frac{d(\rho_{t+\delta t})^{n-1}}{K^n}}{\left(1+\left(\frac{d(\rho_{t+\delta t})}{K}\right)^n\right)^2} d(P_{t+\delta t,t}) - \frac{nR\frac{d(\rho_{t+\delta t})^{n-1}}{K^n}}{\left(1+\left(\frac{d(\rho_{t+\delta t})}{K}\right)^n\right)^2} d(P_{t,t+\delta t}) \right] \\
& + \delta t \left[\frac{R}{1+\left(\frac{d(\rho_{t+\delta t})}{K}\right)^n} + \mu \rho_{t+\delta t} \right].
\end{aligned} \tag{9}$$

As observations are available at discrete time-points $\Delta t, 2\Delta t, ..., T$ we can use Eq (8-9) to propagate the estimates of the mean and covariance of the unobserved states up until the next observation $y_{t+\Delta t}$ and subsequently condition on $y_{t+\Delta t}$ using Kalman update

$$\begin{aligned}
\rho^*_{t-\tau_{\max}+\Delta t:t+\Delta t} &= \rho_{t-\tau_{\max}+\Delta t:t+\Delta t} + C\left(y_{t+\Delta t} - F\rho_s\right) \\
P^*_{t+\Delta t-\tau_m:t+\Delta t} &= P_{t+\Delta t-\tau_m:t+\Delta t} - CFP_{t+\Delta t,t+\Delta t-\tau_m:t+\Delta t}
\end{aligned} \tag{10}$$

where

$$C = P_{t+\Delta t-\tau_m:t+\Delta t,t+\Delta t} F^\intercal (FP_{t+\Delta t}F^\intercal + \Sigma_\eta)^{-1}. \tag{11}$$

The marginal likelihood of parameters θ given observations can be decomposed as $\mathcal{L}(\theta|y_{1:T}) = \pi(y_{1:T}|\theta) = \prod_{t=1}^{T} \pi(y_t|y_{1:t-1}, \theta)$. We have Gaussian errors and $E[y_t|y_{1:t-\Delta t}, \theta] = F\rho_{t-\Delta t+\delta t:t}$, and $\mathrm{Var}(Y_t|Y_{1:t-1}, \theta) = FP_{t-\Delta t+\delta t:t}F^{\intercal} + \sigma_{\eta}^2$. Letting $e_t = y_t - F\rho_{t-\Delta t+\delta t:t}$ we can write the marginal log-likelihood, up to an additive constant, as

$$\log \mathcal{L}(\theta|y_{1:T}) = -\frac{1}{2}\sum_{t=1}^{T}[\log|FP_{t-\Delta t+\delta t:t}F^{\intercal} + \Sigma_{\eta}| + e_t^{\intercal}(FP_{t-\Delta t+\delta t:t}F^{\intercal} + \Sigma_{\eta})^{-1}e_t] + c, \tag{12}$$

where the resulting sum is interpreted as a function of the parameters given the fixed observations.

3 Remarks on studying explant SCN

In making the slices, we routinely use the central and majority part of the SCN at its widest and highest extent. This means that the most anterior and posterior poles are absent. In our broad experience, although not in the current dataset, if we use the tissue from the two poles, the rhythmic properties are the same as in the central majority area. An anonymous referee questioned whether the process of the experiment could obscure normal function in situ. We need to take it ex situ to observe it, and that may affect SCN properties. One definite way in which the process of the experiment could affect normal function in situ is that the retinal input necessary for entrainment of the SCN is absent. As we are interested in the intrinsic, free-running oscillator this absence is, in fact, necessary and helpful. Taking the slice will also deprive the SCN of other neural inputs that mediate the effects of behavioural state on the oscillation but the same point applies here. As for the intrinsic oscillation, we have two means to assess the representative value of the imaged slice. First, it is possible to examine the instantaneous pattern of gene expression across the SCN in tissue sections taken from the brains of animals sampled at different circadian phases. A compilation of such "snap-shots" shows that the oscillatory properties observed in the living slice match the in vivo pattern. While spatial resolution is excellent the latter lacks temporal resolution and is a composite of data from many animals, and not from a single SCN/ single SCN cells monitored continuously through time. Second, methods are now being developed for in vivo imaging using mini microscopes targeted above the SCN of awake, behaving mice and used to record bioluminescent or fluorescent signals. This again shows oscillatory properties in vivo that match what we would predict as an integrated total of the SCN slice data. It does not, however, have any spatial resolution. Together, the datasets available indicate that what we observe in the ex vivo slice replicates properties of the free-running SCN in vivo. All approaches have their particular caveats. Another question, raised by the anonymous referee is whether the spatial distributions of parameters near SCN edges is simply due to there being fewer oscillatory cells? Anatomically, the SCN is tightly defined at its border - the anatomical limits are not graded or diffuse. The SCN cells are tightly packed right up to the border and then give way within a few microns distance to more loosely packed surrounding hypothalamus. When preparing the slices, most of the surrounding non-SCN tissue is trimmed off but not all of it, as we wish to avoid damaging the SCN proper. In our recordings the strength of oscillation of SCN cells close to the border is not different from more centrally located SCN cells.

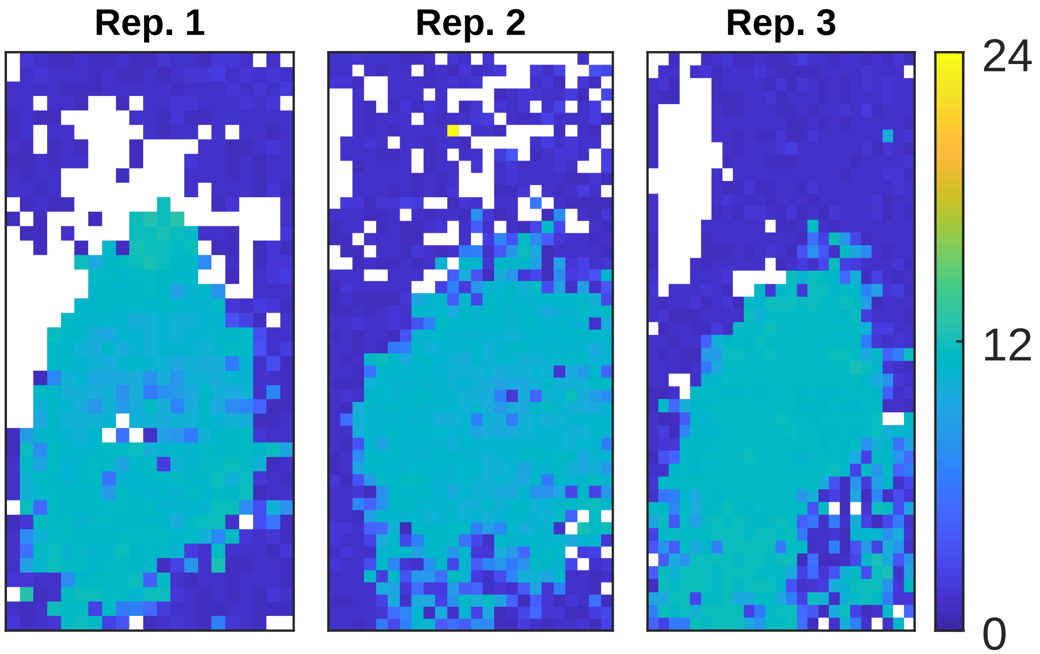

Fig A. Spatial distribution of residual periodicity for locations where 99 % spectral bootstrap confidence intervals have an endpoint in the range 1-30h. Residuals are mostly free of 24 hour periodicity while locations corresponding to SCN tissue typically exhibit additional low-amplitude 12-hour periodicity.

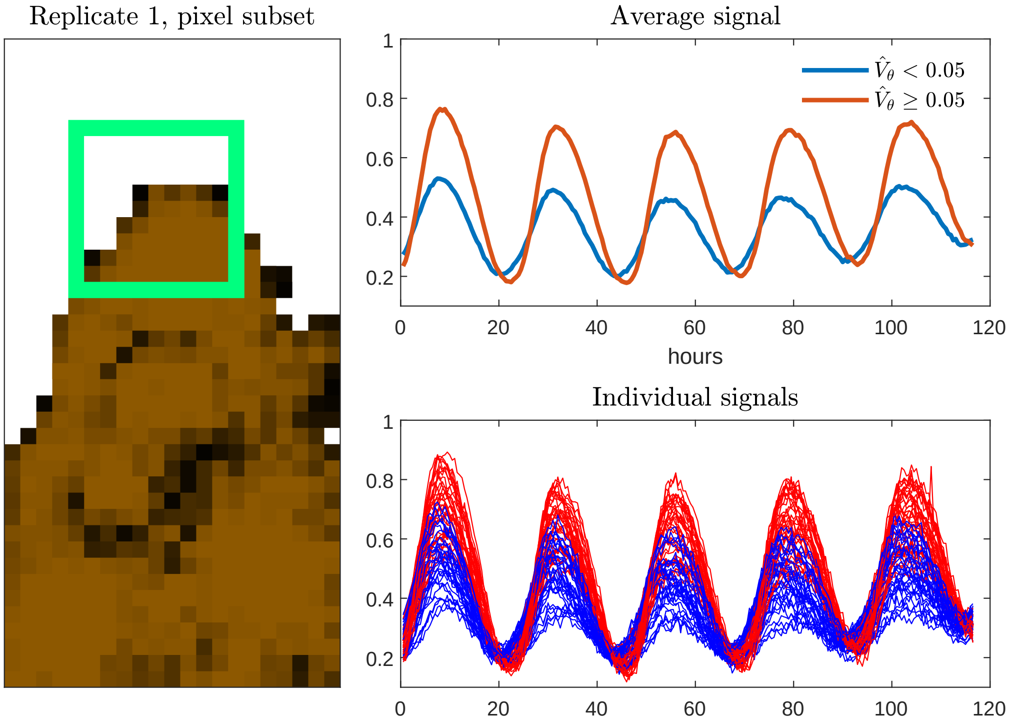

Fig B. Average signal in subset of pixels by $\hat{V}_\theta$ cut-off. Cross section of SCN with selected location marked with a green square. Average and individual signals for subset of central pixels with $\hat{V}_\theta < 0.05$ (blue) and $\hat{V}_\theta \geq 0.05$ (red) over approximately 120 hours. Locations with $\hat{V}_\theta < 0.05$ (blue) typically exhibit lower amplitude oscillations, however substantial overlap between the two regions is evident.

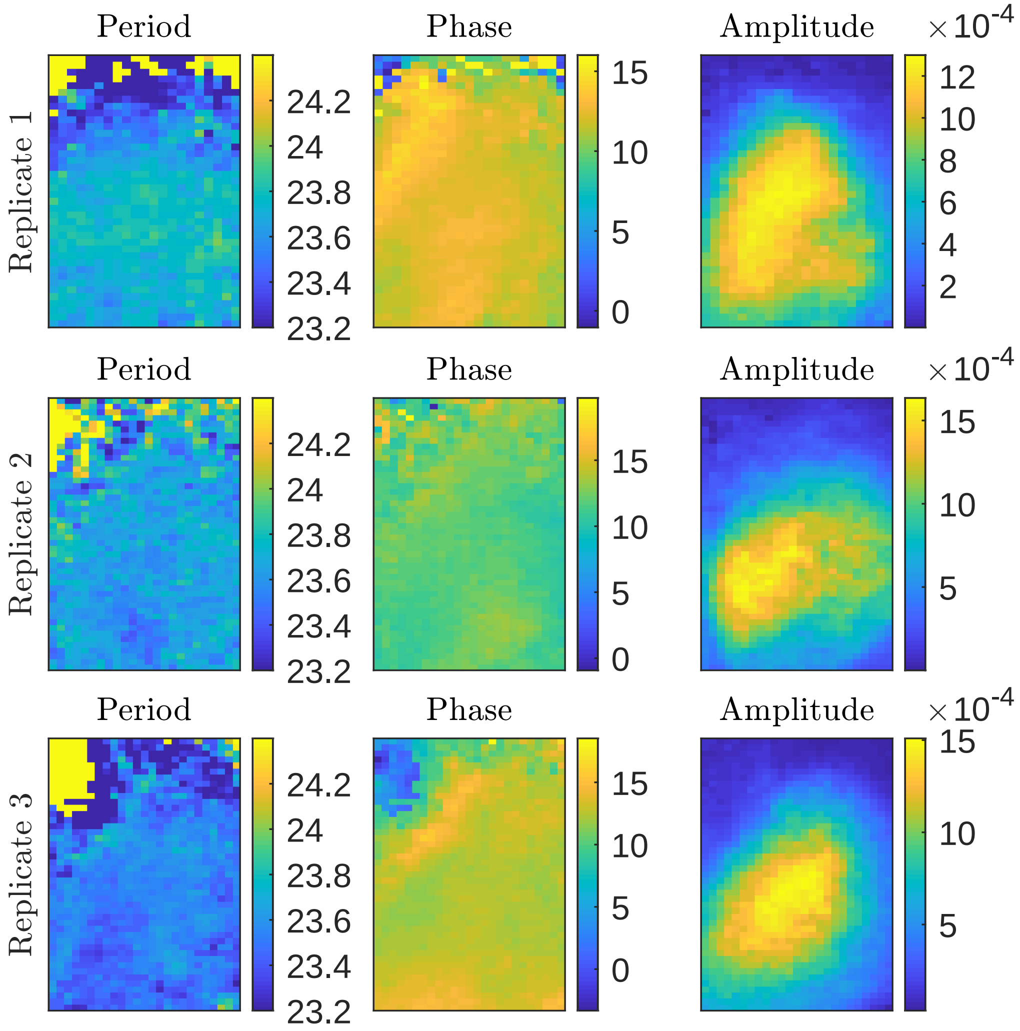

Fig C. Period, phase and amplitude of analysed Cry1-luc data across three replicates. Period and phase of dominant 24h frequency shows little to no spatial structure, apart from top-most edge which is imaged non-tissue. Amplitude is generally higher in central location. This is likely caused by two mechanisms: i) higher concentration of Cry1-luc in SCN proper and ii) the geometry of the tissue sample and experimental procedure whereby these locations are closer to the camera.

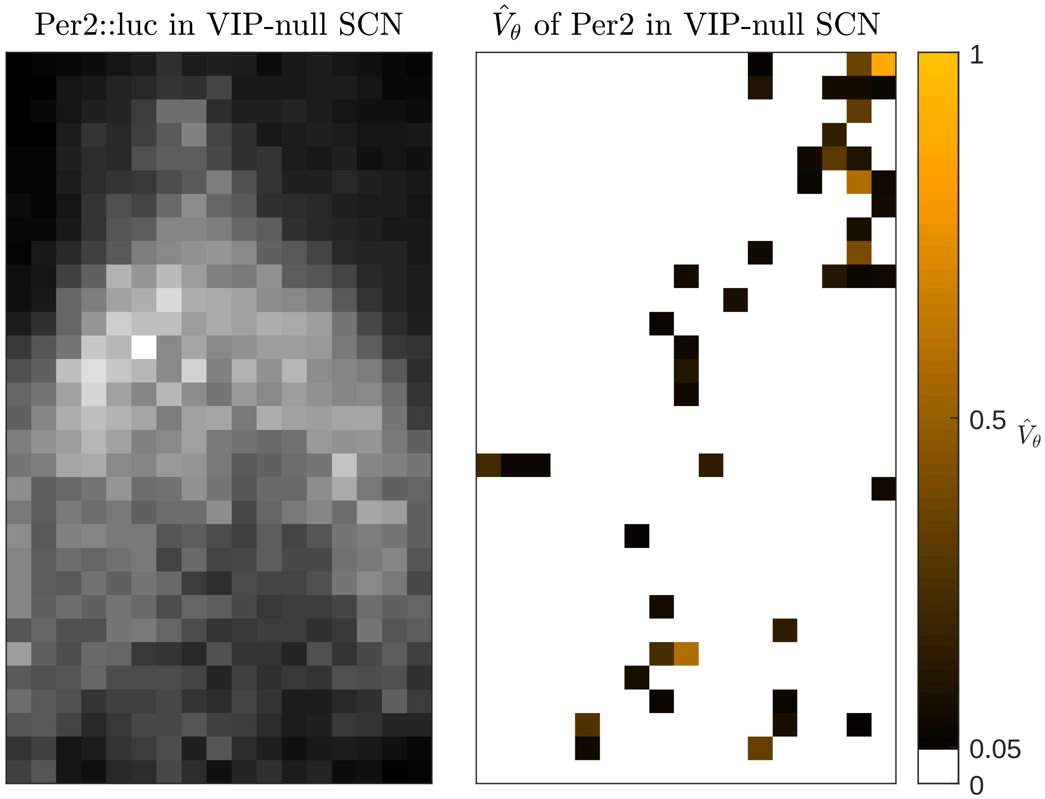

Fig D. Spatial distribution of oscillator robustness, $\hat{V}_\theta$, for Per2:luc in grid of locations across VIP-null SCN tissue, data made available by [3]. Model and parameter estimation are the same as that of the main text. In VIP-null tissue we estimate $\hat{V}_\theta < 0.05$ in most locations across SCN proper.

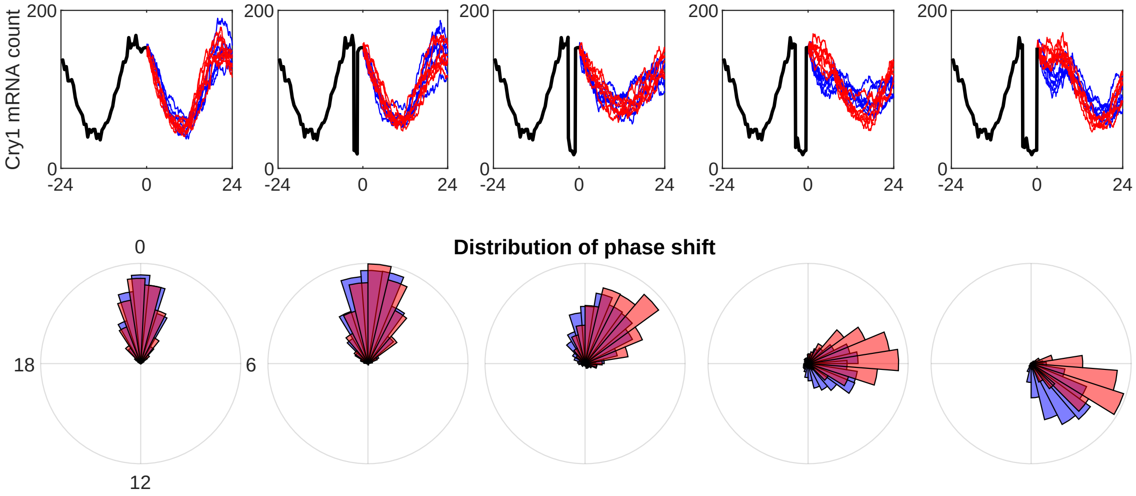

Fig E. Differential responses to large negative perturbations of varying duration. Parameters are set to $R_0 = 50$, $K = 100$, $\mu = 0.25$ for both oscillator types while the Hill coefficient is set to $n = 5.57$ and $n = 3.82$ respectively. Delay mean is set to 9.4 and 9.1, and delay standard deviation is set to 4.2 and 2.2 respectively. Initial data is obtained by scaling the light signal by $\kappa = 2.5 \times 10^{-3}$ which is a typical estimate for SCN tissue. Perturbations are defined as a negative shock of -130 molecules during the timing of the peak Cry1 concentration in the initial data. Trajectories are simulated using an Euler-Maruyama approximation to the model in Equation (1) of the main text with time step set to $dt = 0.1$h. Bootstrap phase distributions are obtained by simulating 2000 paths and calculating the phase of the subsequent five cycles after the visualized paths in the figure.

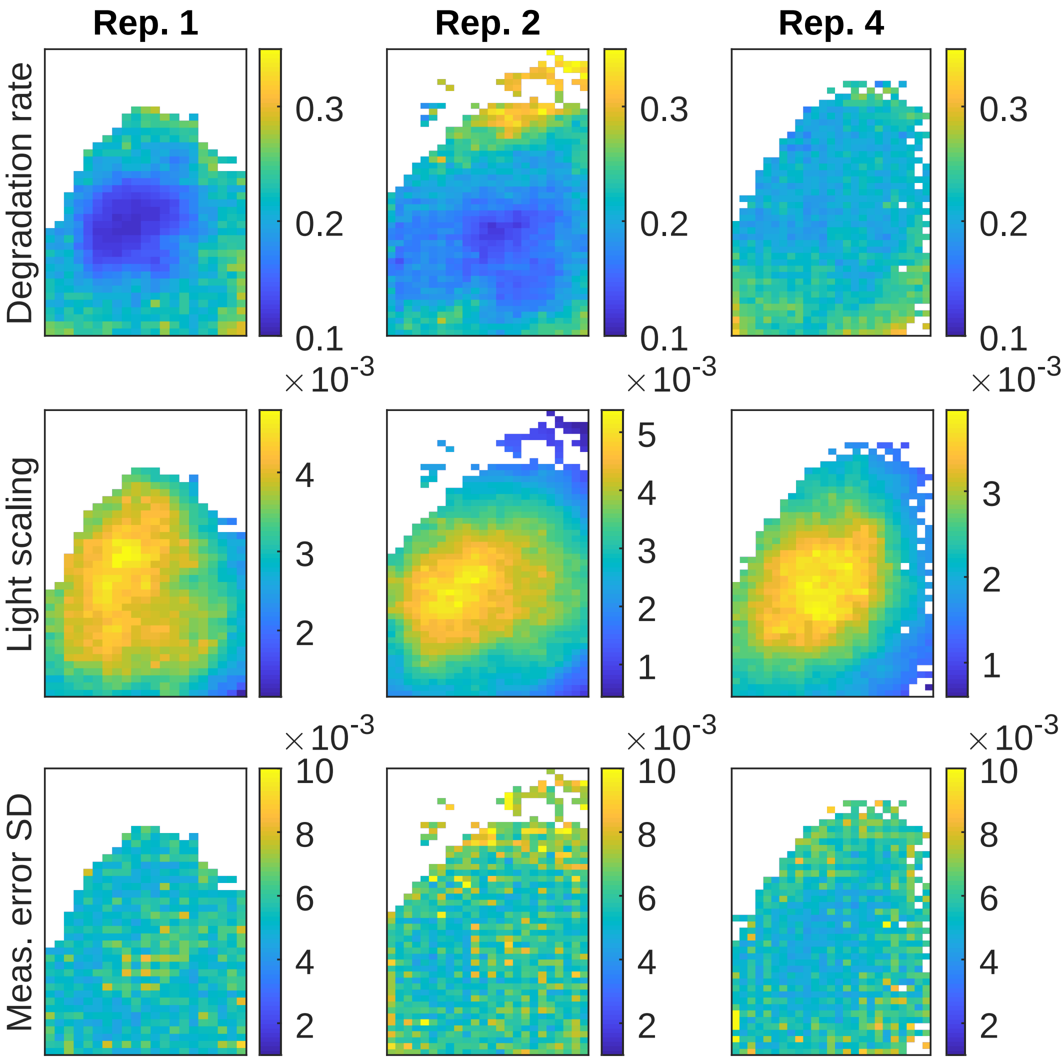

Fig F. Spatial distribution of posterior means for degradation rate (μ), light scaling constant (κ) and measurement error SD (σ_η).

References

1. Smith H. Distributed delay equations and the linear chain trick. In: An introduction to delay differential equations with applications to the life sciences. Springer; 2011. p. 119–130.

2. Mallet-Paret J, Smith HL. The Poincaré-Bendixson theorem for monotone cyclic feedback systems. Journal of Dynamics and Differential Equations. 1990;2(4):367–421.

3. Maywood ES, Chesham JE, O'Brien JA, Hastings MH. A diversity of paracrine signals sustains molecular circadian cycling in suprachiasmatic nucleus circuits. Proceedings of the National Academy of Sciences. 2011;108(34):14306–14311.
